# Association Between Diabetes Mellitus and Cytochrome P450 Family 2 Subfamily C Member 19 (CYP2C19) Loss-of-Function Allele Carriage Among Patients Undergoing Coronary Artery Bypass Grafting: A Cross-Sectional Study

**DOI:** 10.7759/cureus.111991

**Published:** 2026-07-03

**Authors:** Most. Sumaia Bin-Te Shawkat, A.S.M. Hossain Kabir, Istiaq Ahmed, Md. Mohashin Reza, Md. Rezwanul Hoque Bulbul

**Affiliations:** 1 Cardiac Surgery, Dhaka Medical College Hospital, Dhaka, BGD; 2 Cardiac Surgery, Sir Salimullah Medical College Mitford Hospital, Dhaka, BGD; 3 Cardiac Surgery, Bangladesh Medical University, Dhaka, BGD

**Keywords:** clopidogrel resistance, coronary artery bypass grafting, coronary artery disease, cyp2c19 polymorphism, diabetes mellitus, pharmacogenetics

## Abstract

Background: Cytochrome P450 family 2 subfamily C member 19 (CYP2C19) loss-of-function (LOF) polymorphisms reduce the metabolic activation of clopidogrel and contribute to interindividual variability in antiplatelet response. Diabetes mellitus is associated with increased platelet reactivity and adverse cardiovascular outcomes; however, its association with CYP2C19 LOF allele carriage among patients undergoing coronary artery bypass grafting (CABG) remains incompletely understood.

Objective: This study aimed to evaluate the association between diabetes mellitus and CYP2C19 LOF allele carriage among patients undergoing CABG and to describe the distribution of CYP2C19 genotypes and metabolizer phenotypes in this population.

Methods: This analytical cross-sectional study included 100 consecutive patients undergoing elective CABG. CYP2C19 genotyping (*1, *2, *3, and *17 alleles) was performed using real-time polymerase chain reaction. LOF allele carriage was defined as the presence of at least one reduced-function allele (*2 or *3). Because platelet function testing was not performed, the study evaluated genetically predicted reduced clopidogrel metabolism rather than laboratory-confirmed clopidogrel resistance. Associations between diabetes mellitus and LOF allele carriage were examined using multivariable logistic regression adjusted for age, sex, smoking history, hypertension, and dyslipidemia.

Results: The mean age of participants was 58.6 ± 9.3 years, and 72% were male participants. Overall, 46 of 100 patients (46.0%) carried at least one CYP2C19 LOF allele. LOF allele carriage was more common among patients with diabetes mellitus than among those without diabetes (58.0% vs. 34.0%; odds ratio (OR), 2.68; 95% confidence interval (CI), 1.19-6.02; p = 0.016). After adjustment for potential confounders, diabetes mellitus remained independently associated with LOF allele carriage (adjusted OR, 1.95; 95% CI, 1.04-3.65; p = 0.037). Other clinical variables, including age, sex, smoking history, hypertension, and dyslipidemia, were not significantly associated with LOF allele carriage.

Conclusions: In this cross-sectional study, diabetes mellitus was associated with a higher prevalence of CYP2C19 LOF allele carriage among patients undergoing CABG. Because platelet function testing was not performed, these findings reflect genetically predicted reduced clopidogrel metabolism rather than confirmed clopidogrel resistance. Larger prospective studies incorporating platelet function testing and clinical outcome assessment are required to confirm these findings and determine their clinical significance.

## Introduction

Coronary artery disease (CAD) continues to represent a major public health challenge and remains among the most common causes of cardiovascular morbidity and mortality worldwide. Despite advances in preventive cardiology, medical management, and surgical revascularization, ischemic cardiovascular events continue to occur frequently in patients with established atherosclerotic disease [1]. Platelet activation and thrombus formation play central roles in the pathogenesis of acute coronary syndromes and other adverse cardiovascular outcomes, making antiplatelet therapy an essential component of contemporary cardiovascular care [2].

Among currently available antiplatelet agents, clopidogrel remains widely prescribed because of its proven efficacy, favorable safety profile, broad availability, and relatively low cost. The drug is commonly used in patients undergoing coronary revascularization procedures, including coronary artery bypass grafting (CABG). However, considerable interindividual variability exists in the therapeutic response to clopidogrel. While many patients achieve adequate platelet inhibition, others exhibit reduced responsiveness, potentially increasing the risk of thrombotic complications such as myocardial infarction, graft occlusion, ischemic stroke, and cardiovascular death [3,4].

The antiplatelet effect of clopidogrel depends on a complex metabolic activation process within the liver. As an inactive prodrug, clopidogrel requires conversion to its active metabolite through multiple cytochrome P450 enzymes, among which cytochrome P450 family 2 subfamily C member 19 (CYP2C19) is particularly important. Functional genetic variants of the CYP2C19 gene influence enzyme activity and may alter the extent of active metabolite formation. Reduced-function alleles, most notably CYP2C192 and CYP2C193, are associated with impaired metabolic activation of clopidogrel and diminished platelet inhibition. In contrast, the CYP2C19*17 allele has been associated with enhanced enzymatic activity and increased drug responsiveness [5,6].

Accumulating evidence indicates that carriers of CYP2C19 loss-of-function (LOF) alleles experience reduced antiplatelet efficacy and may be at greater risk of adverse cardiovascular outcomes when treated with standard clopidogrel therapy [7,8]. These observations have contributed to the growing field of cardiovascular pharmacogenomics, which seeks to optimize drug therapy by incorporating genetic information into clinical decision-making. Current recommendations from the Clinical Pharmacogenetics Implementation Consortium (CPIC) support consideration of alternative antiplatelet strategies for selected patients carrying CYP2C19 LOF variants, particularly when the anticipated risk of ischemic events is high [9].

In addition to genetic influences, diabetes mellitus is recognized as an important determinant of platelet function and thrombotic risk. Chronic hyperglycemia promotes endothelial dysfunction, oxidative stress, inflammatory activation, and abnormalities in platelet signaling pathways. These changes contribute to increased platelet reactivity and may reduce the effectiveness of conventional antiplatelet therapy [10]. Previous investigations have shown that patients with diabetes often exhibit higher residual platelet activity during clopidogrel treatment compared with nondiabetic individuals, suggesting that metabolic disturbances may further modify treatment response [11,12].

The interaction between diabetes mellitus and CYP2C19 genetic polymorphisms may be particularly relevant in patients undergoing CABG. Maintenance of graft patency and prevention of postoperative ischemic complications depend heavily on effective antiplatelet therapy. Identification of individuals who carry CYP2C19 LOF alleles may facilitate more personalized treatment approaches and improve perioperative risk stratification. Although numerous studies have evaluated CYP2C19 pharmacogenetics in Western and East Asian populations, evidence from Bangladesh and other South Asian countries remains limited. Furthermore, data regarding the combined influence of diabetes mellitus and CYP2C19 polymorphisms among patients undergoing CABG are scarce.

Therefore, the present study was undertaken to determine the prevalence of CYP2C19 polymorphisms associated with genetically predicted clopidogrel resistance among patients undergoing CABG and to compare these findings between diabetic and nondiabetic individuals. In addition, the study aimed to characterize the distribution of CYP2C19 genotypes and to evaluate the association between diabetes mellitus and genetically predicted clopidogrel resistance in this population.

## Materials and methods

Study design and setting

This analytical comparative cross-sectional study was conducted in the Department of Cardiac Surgery at Bangabandhu Sheikh Mujib Medical University, Dhaka, Bangladesh, between August 2022 and February 2024. The study was designed to evaluate the relationship between CYP2C19 genetic polymorphisms and genetically predicted clopidogrel resistance among diabetic and nondiabetic patients with angiographically confirmed CAD undergoing elective CABG. Because platelet function testing was not available, the study assessed genetically predicted reduced clopidogrel metabolism based on CYP2C19 genotype rather than laboratory-confirmed clopidogrel resistance.

Ethical considerations

Ethical approval was obtained from the Institutional Review Board of Bangabandhu Sheikh Mujib Medical University before the commencement of the study (approval no. BSMMU/2023/5613). All participants provided written informed consent before inclusion in the investigation. The study adhered to the principles of the Declaration of Helsinki and all applicable ethical standards governing human research.

Study population

Patients with angiographically confirmed CAD who were scheduled for elective CABG during the study period were screened consecutively for eligibility.

Inclusion criteria

Eligible participants were adults aged 18 years or older with angiographically confirmed CAD who were scheduled for elective CABG and had received clopidogrel at a dose of 75 mg daily for at least seven days before surgery. Only individuals who provided written informed consent were included.

Exclusion criteria

Patients were excluded if they had received prasugrel or ticagrelor within the preceding 30 days, had known platelet disorders, active bleeding, severe hepatic dysfunction, end-stage renal disease, active malignancy, autoimmune disease, or required additional cardiac surgical procedures besides CABG. Individuals who were unable or unwilling to provide informed consent were also excluded.

Sample size calculation

The sample size was determined using the formula for comparison of two independent proportions as described by Charan and Biswas [13]:

\(n = \frac{\left(Z_{\alpha/2} + Z_{\beta}\right)^2 \left\{P_1(1-P_1) + P_2(1-P_2)\right\}} {\left(P_1 - P_2\right)^2}\)

where n is the required sample size per group; Zα/2 is 1.96 for a 95% confidence level; Zβ is 0.84, corresponding to 80% statistical power; P₁ is the expected prevalence in diabetic patients; and P₂ is the expected prevalence in nondiabetic patients.

The expected prevalence of reduced clopidogrel responsiveness among diabetic and nondiabetic patients was estimated at 55% and 30%, respectively. The prevalence assumptions were based on published estimates of reduced clopidogrel responsiveness among diabetic and nondiabetic populations [14,15]. Using these assumptions, the minimum required sample size was calculated to be 46 participants in each group. To compensate for potential exclusions and incomplete data, 50 patients were recruited into each group, resulting in a final study population of 100 participants.

Sampling technique

A consecutive sampling technique was employed. Eligible patients were enrolled sequentially until the target sample size was achieved. Fifty patients with diabetes mellitus constituted the diabetic group, while 50 patients without diabetes mellitus constituted the nondiabetic group.

Clinical assessment and data collection

Baseline demographic and clinical information was collected using a structured data collection form. Variables included age, sex, body mass index, smoking history, hypertension, dyslipidemia, family history of CAD, previous myocardial infarction, previous coronary intervention, duration of diabetes mellitus, and glycated hemoglobin level.

Coronary angiographic findings were reviewed and categorized as single-vessel disease, double-vessel disease, or triple-vessel disease according to the extent of coronary artery involvement.

Diabetes mellitus was defined as a documented physician diagnosis, current use of glucose-lowering medication, fasting plasma glucose ≥126 mg/dL, HbA1c ≥6.5%, or according to the American Diabetes Association diagnostic criteria [14].

Blood sample collection and deoxyribonucleic acid extraction

Prior to surgery, 5 mL of peripheral venous blood was collected under aseptic conditions into ethylenediaminetetraacetic acid-containing tubes. Samples were transported immediately to the molecular genetics laboratory for analysis.

Genomic DNA was isolated from peripheral leukocytes utilizing a standardized commercial extraction system following the recommended laboratory protocol. Spectrophotometric evaluation was performed to verify DNA quantity and purity prior to downstream amplification analyses.

CYP2C19 genotyping

Genotyping of the CYP2C19 alleles (*1, *2, *3, and *17) was performed using real-time polymerase chain reaction (PCR) with allele-specific hydrolysis (TaqMan®; Applied Biosystems, Thermo Fisher Scientific, Waltham, MA) fluorescent probes. Amplification was carried out using a QuantStudio™ 5 Real-Time PCR System (Applied Biosystems, Thermo Fisher Scientific) and a commercially available CYP2C19 genotyping assay according to the manufacturer's instructions. The PCR reaction was performed in a total volume of 20 μL, containing genomic DNA, TaqMan Genotyping Master Mix, allele-specific primers, and fluorescent probes.

Thermal cycling conditions consisted of an initial denaturation at 95°C for 10 minutes, followed by 40 cycles of denaturation at 95°C for 15 seconds and annealing/extension at 60°C for 60 seconds. Genotypes were assigned automatically using the instrument's allelic discrimination software and were independently reviewed by two investigators. Positive and negative controls were included in each PCR run, and samples with ambiguous amplification curves were reanalyzed before final genotype assignment. DNA concentration and purity were assessed spectrophotometrically prior to amplification.

Classification of metabolizer phenotypes

Phenotypes were classified according to the CPIC guidelines. CYP2C19 *1/*1 was classified as a normal metabolizer; *1/*2 and *1/*3 as intermediate metabolizers; *2/*2, *2/*3, and *3/*3 as poor metabolizers; *1/*17 as a rapid metabolizer; and *17/*17 as an ultrarapid metabolizer. For the primary analysis, CYP2C19 LOF allele carriage was defined as the presence of at least one reduced-function allele (*2 or *3), regardless of zygosity. Accordingly, participants with genotypes *1/*2, *1/*3, *2/*2, *2/*3, or *3/*3 were classified as LOF allele carriers, whereas those with *1/*1, *1/*17, or *17/*17 were classified as non-LOF carriers.

Outcome measures

The primary outcome was CYP2C19 LOF allele carriage, representing genetically predicted reduced clopidogrel metabolism based on CYP2C19 genotype. Because platelet function testing was not performed, the study evaluated genetically predicted reduced clopidogrel metabolism rather than laboratory-confirmed clopidogrel resistance.

The secondary outcomes of the study were to describe the distribution of CYP2C19 genotypes (*1/*1, *1/*2, *1/*17, *2/*3, and other detected variants) among patients undergoing elective CABG, compare the frequency of CYP2C19 LOF allele carriage between patients with and without diabetes mellitus, characterize the distribution of CYP2C19 metabolizer phenotypes (normal, intermediate, poor, rapid, and ultrarapid metabolizers) according to CPIC recommendations, and estimate the association between diabetes mellitus and CYP2C19 LOF allele carriage after adjustment for selected demographic and clinical covariates (age, sex, smoking status, hypertension, and dyslipidemia).

Statistical analysis

Statistical analyses were performed using IBM Statistical Package for the Social Sciences, version 26.0 (IBM Corp., Armonk, NY). Continuous variables were assessed for normality using the Shapiro-Wilk test and are presented as mean ± standard deviation. Categorical variables are presented as frequencies and percentages.

Comparisons between diabetic and nondiabetic patients were performed using the independent-samples Student's t-test for normally distributed continuous variables and Pearson's chi-square test or Fisher's exact test for categorical variables, as appropriate.

The primary analysis evaluated the association between diabetes mellitus and CYP2C19 LOF allele carriage. Multivariable binary logistic regression was used to estimate the adjusted association between diabetes mellitus and LOF allele carriage while controlling for age, sex, smoking status, hypertension, and dyslipidemia. CYP2C19 LOF allele carriage (yes/no) was specified as the dependent variable. Clinical covariates were selected a priori based on their potential to confound the observed association and were included to obtain adjusted estimates rather than to identify biological predictors of inherited genotype. Adjusted odds ratios (ORs) with 95% confidence intervals (CIs) were reported.

Multicollinearity was evaluated using variance inflation factors (VIFs) and tolerance statistics before model fitting. Model calibration was assessed using the Hosmer-Lemeshow goodness-of-fit test, and model discrimination was summarized using Nagelkerke's R². Complete-case analysis was performed because no missing data were identified. All statistical tests were two-sided, and a p value of <0.05 was considered statistically significant.

## Results

A total of 100 patients with angiographically confirmed CAD undergoing elective CABG were included in the study. The study population comprised 50 patients with diabetes mellitus and 50 without. Complete demographic, clinical, laboratory, and genetic data were available for all participants, and no enrolled patient was excluded from the final analysis.

The mean age of the overall study population was 58.6 ± 9.3 years. Patients in the diabetic group were slightly older than those in the nondiabetic group (59.8 ± 9.1 vs. 57.4 ± 9.5 years), although the difference did not reach statistical significance (p = 0.18). Male patients accounted for 72 of 100 (72.0%) participants. No statistically significant differences were observed between the groups regarding age, sex distribution, body mass index, smoking history, dyslipidemia, or family history of CAD. Hypertension was significantly more prevalent among diabetic patients than nondiabetic patients (39 (78.0%) vs. 28 (56.0%), p = 0.02) (Table 1).

**Table 1:** Baseline demographic and clinical characteristics of the study population Data are presented as mean ± SD for continuous variables and number (percentage) for categorical variables. Continuous variables were compared using the independent-samples Student's t-test, whereas categorical variables were analyzed using Pearson's chi-square (χ²) test. A two-tailed p value <0.05 was considered statistically significant ^*^Statistically significant difference between the groups (p < 0.05) SD: Standard deviation

Variable	Diabetes mellitus (n = 50)	Nondiabetes mellitus (n = 50)	Test statistic	p value
Age (years), mean ± SD	59.8 ± 9.1	57.4 ± 9.5	t = 1.29	0.18
Male sex, n (%)	35 (70.0)	37 (74.0)	χ² = 0.20	0.65
Body mass index (kg/m²), mean ± SD	27.6 ± 3.8	26.4 ± 3.4	t = 1.66	0.08
Smoking history, n (%)	31 (62.0)	34 (68.0)	χ² = 0.40	0.53
Hypertension, n (%)	39 (78.0)	28 (56.0)	χ² = 5.47	0.02^*^
Dyslipidemia, n (%)	33 (66.0)	24 (48.0)	χ² = 3.31	0.07
Family history of coronary artery disease, n (%)	17 (34.0)	12 (24.0)	χ² = 1.22	0.27

The distribution of CYP2C19 genotypes is summarized in Table 2. The most frequently observed genotype was CYP2C19 *1/*2 (39.0%), followed by CYP2C19 *1/*1 (34.0%), CYP2C19 *1/*17 (20.0%), CYP2C19 *2/*3 (5.0%), and CYP2C19 *1/*3 (2.0%). No participants carried the *2/*2, *3/*3, or *17/*17 genotypes. Overall, 46% (46/100) of participants carried at least one CYP2C19 LOF allele (*2 or *3), representing genetically predicted reduced clopidogrel metabolism (Table 2).

**Table 2:** Distribution of CYP2C19 genotypes among diabetic and nondiabetic patients Data are presented as n (%). Genotype frequencies are reported descriptively according to diabetes status. Because of the small number of patients carrying the CYP2C19*2/*3 genotype, formal statistical comparisons of genotype distribution between diabetic and nondiabetic groups were not emphasized. No participants carried CYP2C19 *2/*2, *3/*3, or *17/*17 genotypes. Two participants carried the *1/*3 genotype CYP2C19: cytochrome P450 family 2 subfamily C member 19

Genotype	Diabetes (n = 50)	Nondiabetes (n = 50)	Total (n = 100)
*1/*1	13 (26.0)	21 (42.0)	34 (34.0)
*1/*2	22 (44.0)	17 (34.0)	39 (39.0)
*1/*3	2 (4.0)	0 (0.0)	2 (2.0)
*2/*3	3 (6.0)	2 (4.0)	5 (5.0)
*1/*17	10 (20.0)	10 (20.0)	20 (20.0)

CYP2C19 LOF allele carriage (genetically predicted reduced clopidogrel metabolism) was more frequently observed among patients with diabetes mellitus than among those without diabetes (58.0% vs. 34.0%). Unadjusted analysis demonstrated that diabetes mellitus was significantly associated with CYP2C19 LOF allele carriage (OR, 2.68; 95% CI, 1.19-6.02; p = 0.016), indicating that patients with diabetes had approximately 2.7-fold higher odds of carrying a CYP2C19 LOF allele than patients without diabetes. Because platelet function testing was not performed, these findings reflect an association with genetically predicted reduced clopidogrel metabolism rather than laboratory-confirmed clopidogrel resistance (Table 3).

**Table 3:** Association between diabetes mellitus and genetically predicted clopidogrel resistance Data are presented as n (%). Group differences were analyzed using Pearson’s chi-square (χ²) test. ORs with 95% CIs were calculated to estimate the strength of association ^*^Statistically significant association (p < 0.05) CI: confidence interval; OR: odds ratio

Group	Genetically predicted clopidogrel resistance, n (%)	No genetically predicted clopidogrel resistance, n (%)	OR (95% CI)	χ²	p value
Diabetes mellitus (n = 50)	29 (58.0)	21 (42.0)	2.68 (1.19-6.02)	5.79	0.016^*^
Nondiabetes mellitus (n = 50)	17 (34.0)	33 (66.0)	Reference	-	-

To estimate the association between diabetes mellitus and CYP2C19 LOF allele carriage while accounting for potential confounding, a multivariable logistic regression analysis was performed, adjusting for age, sex, smoking status, hypertension, and dyslipidemia. After adjustment, diabetes mellitus remained significantly associated with CYP2C19 LOF allele carriage (adjusted OR, 1.95; 95% CI, 1.04-3.65; p = 0.037). None of the other clinical variables showed statistically significant associations with LOF allele carriage. The regression model demonstrated acceptable calibration according to the Hosmer-Lemeshow goodness-of-fit test (χ² = 5.12, p = 0.74) and modest explanatory ability (Nagelkerke R² = 0.18). VIFs ranged from 1.06 to 1.42, indicating no evidence of clinically important multicollinearity. Because the CYP2C19 genotype is inherited and fixed at conception, these findings should be interpreted as adjusted statistical associations between clinical characteristics and LOF allele carriage within the study population rather than evidence that these clinical variables influence or determine genotype (Table 4).

**Table 4:** Multivariable logistic regression estimating the association between diabetes mellitus and CYP2C19 LOF allele carriage after adjustment for clinical covariates Data are presented as regression coefficient (β), SE, adjusted OR, 95% CI, Wald χ² statistic, and p value. Multivariable logistic regression was performed to estimate the adjusted association between diabetes mellitus and CYP2C19 LOF allele carriage while controlling for age, sex, smoking history, hypertension, and dyslipidemia. CYP2C19 LOF allele carriage (yes/no) was specified as the dependent variable. Because the CYP2C19 genotype is inherited, the reported associations should not be interpreted as evidence that clinical variables biologically predict or determine genotype. A two-tailed p value of <0.05 was considered statistically significant CYP2C19: cytochrome P450 family 2 subfamily C member 19; SE: standard error; OR: odds ratio; CI: confidence interval; LOF: loss-of-function ^*^Statistically significant association (p < 0.05)

Variable	Regression coefficient (β)	Adjusted OR	95% CI	Wald chi-square statistic (χ²)	p value
Diabetes mellitus	0.67 (0.32)	1.95	1.04-3.65	4.35	0.037^*^
Age (per year increase)	0.01 (0.02)	1.01	0.97-1.05	0.32	0.57
Male sex	0.21 (0.41)	1.23	0.55-2.76	0.26	0.61
Smoking history	0.18 (0.38)	1.20	0.57-2.52	0.23	0.63
Hypertension	0.27 (0.35)	1.31	0.66-2.61	0.60	0.44
Dyslipidemia	0.15 (0.34)	1.16	0.59-2.27	0.19	0.66

## Discussion

This study evaluated the association between diabetes mellitus and CYP2C19 LOF allele carriage among patients undergoing elective CABG. Overall, 46% of participants carried at least one CYP2C19 LOF allele, and diabetic patients demonstrated a significantly higher prevalence of LOF allele carriage than nondiabetic patients. After statistical adjustment for potential confounders, diabetes mellitus remained associated with CYP2C19 LOF allele carriage. Because the CYP2C19 genotype is inherited and fixed before birth, this association should be interpreted as a statistical observation within this study population rather than evidence that diabetes influences genotype.

The prevalence of CYP2C19 LOF allele carriage observed in the present study is consistent with previous reports from Asian populations, in which CYP2C19 *2 is the predominant reduced-function allele and accounts for substantial interindividual variability in clopidogrel metabolism [14,15]. Carriers of CYP2C19 LOF alleles exhibit reduced biotransformation of clopidogrel into its active metabolite, resulting in diminished platelet inhibition compared with non-carriers [16,17]. Based on this evidence, the CPIC recommends consideration of alternative P2Y12 inhibitors in selected intermediate and poor metabolizers when clinically appropriate [9]. Our findings demonstrate that nearly one-half of patients undergoing CABG carried a CYP2C19 LOF allele, highlighting the potential importance of pharmacogenetic testing in this population.

A significant association was observed between diabetes mellitus and CYP2C19 LOF allele carriage. Importantly, this finding should not be interpreted as evidence that diabetes influences or causes CYP2C19 genetic variation because genotype is inherited and remains constant throughout life. Rather, diabetic patients in this study had a higher prevalence of LOF allele carriage than nondiabetic patients. The biological explanation for this observation cannot be determined from the present cross-sectional study and may reflect population-specific genetic characteristics, sampling variability, or residual confounding. Confirmation in larger multicenter studies involving diverse populations is therefore warranted.

Diabetes mellitus has consistently been associated with increased platelet reactivity, enhanced platelet turnover, endothelial dysfunction, and reduced responsiveness to antiplatelet therapy [18,19]. However, these mechanisms are biologically distinct from inherited CYP2C19 polymorphisms. The present study did not evaluate platelet aggregation or pharmacodynamic response to clopidogrel; therefore, the observed association between diabetes mellitus and CYP2C19 LOF allele carriage should not be interpreted as evidence of impaired clinical response to clopidogrel. Future studies combining pharmacogenetic testing with platelet function assays and clinical outcome measures would provide a more comprehensive assessment of antiplatelet responsiveness after CABG [20,21].

After adjustment for age, sex, smoking status, hypertension, and dyslipidemia, diabetes mellitus remained statistically associated with CYP2C19 LOF allele carriage. The adjustment variables were included to control for potential confounding rather than to identify biological predictors of genotype. Because the CYP2C19 genotype is inherited and fixed before birth, these findings should be interpreted as statistical associations observed within this study population and not as evidence that diabetes mellitus or other clinical characteristics influence genetic variation.

The present study has several strengths. It is among the first investigations evaluating CYP2C19 polymorphisms in patients undergoing CABG in Bangladesh and provides population-specific data regarding CYP2C19 genotype distribution. Standardized molecular genotyping methods were employed, and multivariable analyses were performed to adjust for important demographic and clinical covariates.

Several limitations should be acknowledged. First, the cross-sectional design precludes causal inference. Second, platelet function testing was not performed; therefore, the study evaluated genetically predicted reduced clopidogrel metabolism rather than confirmed clopidogrel resistance. Third, the relatively small sample size and limited number of participants within some genotype subgroups may have reduced statistical precision and increased the possibility of overfitting in the multivariable model. Fourth, Hardy-Weinberg equilibrium analysis and detailed genotyping quality-control procedures were not originally reported. Finally, clinical outcomes such as graft patency, ischemic events, and bleeding were not assessed.

Future multicenter prospective studies incorporating comprehensive CYP2C19 genotyping, platelet function testing, and long-term clinical outcome assessment are needed to determine whether genotype-guided antiplatelet therapy improves outcomes among patients undergoing CABG.

## Conclusions

This study demonstrated a high prevalence of CYP2C19 LOF polymorphisms among patients undergoing elective CABG. Diabetic patients had a significantly higher frequency of CYP2C19 LOF alleles and genetically predicted clopidogrel resistance than nondiabetic patients. These findings indicate an association between diabetes mellitus and genetically predicted clopidogrel resistance in this study population. The observed pharmacogenetic variability may have implications for clopidogrel metabolism; however, because platelet function testing was not performed and the study was cross-sectional, no conclusions can be drawn regarding actual clopidogrel responsiveness or clinical outcomes. Larger prospective studies incorporating platelet function testing and clinical outcome assessment are warranted to further evaluate the clinical significance of these findings.
